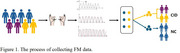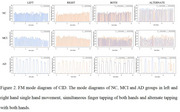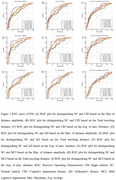# The role of finger movements in the diagnosis of cognitive impairment

**DOI:** 10.1002/alz70860_104152

**Published:** 2025-12-23

**Authors:** Yingren Mai, Zhiyu Cao, Qun Yu, Jun Liu

**Affiliations:** ^1^ The Second Affiliated Hospital of Guangzhou Medical University, Guangzhou, Guangdong, China; ^2^ Department of Neurology, the Second Affiliated Hospital of Guangzhou Medical University, Institute of Neuroscience, the Second Affiliated Hospital of Guangzhou Medical University, Guangzhou, Guangdong, China

## Abstract

**Background:**

Cognitive impairment diseases (CID) are a major public health issue in an aging society, lacking effective cure methods. Early screening is the key to treatment. It is unclear whether digital finger motion (FM) can serve as an early screening tool for CID.

**Method:**

This study included 120 participants, including 60 cognitively normal and 120 CID. Collect digital indicators of FM and analyze the key features and diagnostic accuracy of FM in CID.

**Result:**

A total of 248 FM features were analyzed. There were significant differences (*p* <0.05) between the CID group and the cognitively normal group in 92 key parameters. Of these, the Max of distance amplitude, Avg. of max. distance and Avg. of max. in extending movement velocity were significantly correlated with plasma phosphorylated tau 217 level. The accuracy of identifying CID based on the Max of distance amplitude of a single indicator is 0.800. The accuracy of distinguishing AD by the Avg. of max. distance is 0.865.

**Conclusion:**

Identifying the occurrence of CID requires more objective and accurate selection of digital indicators of FM compared to neuropsychological screening assessment scales.